# Symptom structure of PTSD: support for a hierarchical model separating core PTSD symptoms from dysphoria

**DOI:** 10.3402/ejpt.v3i0.17580

**Published:** 2012-12-13

**Authors:** Arthur R. Rademaker, Agnes van Minnen, Freek Ebberink, Mirjam van Zuiden, Muriel A. Hagenaars, Elbert Geuze

**Affiliations:** 1 Research Centre, Military Mental Healthcare, Utrecht, the Netherlands; 2 Overwaal Centre for Anxiety Disorders, Radboud University Nijmegen, Nijmegen, the Netherlands; 3 Department of Clinical, Health, and Neuropsychology, Leiden University, Leiden, the Netherlands; 4 Centre for Psychological Trauma, Academic Medical Center, Amsterdam, the Netherlands; 5 Department of Psychiatry, Rudolf Magnus Institute of Neuroscience, Utrecht University Medical Centre, Utrecht, the Netherlands

**Keywords:** PTSD, confirmatory factor analysis, trauma, distress disorders, dysphoria

## Abstract

**Background:**

As of yet, no collective agreement has been reached regarding the precise factor structure of posttraumatic stress disorder (PTSD). Several alternative factor-models have been proposed in the last decades.

**Objective:**

The current study examined the fit of a hierarchical adaptation of the Simms et al. (2002) dysphoria model and compared it to the fit of the PTSD model as depicted in the *Diagnostic and Statistical Manual for Mental Disorders*, Fourth Edition (*DSM-IV*), a correlated four-factor emotional numbing, and a correlated four-factor dysphoria model.

**Methods:**

Data were collected using the Clinician-Administered PTSD Scale in a mixed-trauma sample of treatment-seeking PTSD patients (*N*=276).

**Results:**

All examined models provided superior fit to the three-factor model of *DSM-IV*. The hierarchical four-factor solution provided a better fit than competing models.

**Conclusion:**

The present study provides empirical support for a conceptualization of PTSD that includes a higher-order PTSD factor that encompasses re-experiencing, arousal, and effortful avoidance sub-factors and a dysphoria factor.

Individuals who are exposed to extreme or prolonged stress are at risk of developing mental disorders such as posttraumatic stress disorder (PTSD). In the latest revision of the Diagnostic and Statistical manual for Mental disorders (*DSM-IV*-TR; American Psychiatric Association, 2000), PTSD is categorized as an anxiety disorder. Symptoms are organized into three different clusters: re-experiencing (B criteria), avoidance and numbing (C criteria), and hyperarousal (D criteria). However, the nosologic validity of PTSD, including the diagnostic criteria as formulated in DSM, has raised considerable debate (Spitzer, First, & Wakefield, 2007). Issues concerning the diagnosis include (but are not limited to) marked heterogeneity in symptom patterns across individuals diagnosed with PTSD (Foa, Riggs, & Gershuny, 1995), as well as high comorbidity and symptom overlap with other mental disorders (Deering, Glover, Ready, Eddleman, & Alarcon, 1996; Spitzer et al., 2007). Moreover, attempts to provide empirical support for specific pathogenic mechanisms underlying PTSD have yielded equivocal results at best (Rosen & Lilienfeld, 2008).

The marked comorbidity of PTSD with other disorders and the lack of compelling evidence for a specific pathogenesis and etiology of PTSD may imply that there are, in fact, multiple psychopathological dimensions underlying the PTSD construct. Each of these underlying dimensions is potentially related to distinct pathogenic processes. Structural studies may help to identify the underlying (latent) components and to determine which of these are specific to PTSD, versus those that would explain existing comorbidity with other (mood and anxiety) disorders (Simms, Watson, & Doebbeling, 2002). For instance, it has previously been proposed that PTSD shares a broad, non-specific factor with “distress” disorders (major depressive disorder [MDD], generalized anxiety disorder [GAD], dysthymic disorder [DD]), as well as with other emotional/internalizing disorders (i.e., fear disorders and bipolar disorders), but that it is distinguishable from these disorders by one or more specific (lower-level) components (Watson, 2005). Identification of shared versus specific components of PTSD would facilitate research into the (neurobiological) basis of PTSD symptoms. Moreover, identification of psychopathological constructs underlying PTSD could aid in diagnosis and effective treatment.

A considerable number of studies examined the latent structure of PTSD using exploratory or Confirmatory Factor Analysis (CFA) (King, King, & Orazem, 2006). On the whole, the majority of studies indicate that the *DSM-IV* model does not adequately represent the latent structure of PTSD. Several models have been proposed in the last decades, including two- (Asmundson, Wright, McCreary, & Pedlar, 2003; Buckley, Blanchard, & Hickling, 1998; Taylor, Koch, Kuch, Crockett, & Passey, 1998), three- (Anthony, Lonigan, & Hecht, 1999; Foa et al., 1995), four- (Asmundson et al., 2000; Baschnagel, O'Connor, Colder, & Hawk, 2005; King, Leskin, King, & Weathers, 1998; Simms et al., 2002), and five-factor solutions (Dragan, Lis-Turlejska, Popiel, Szumial, & Dragan, 2012; Elhai et al., 2011; Morina et al., 2010; Watson et al., 1991). Models that have been replicated most consistently across studies are the four-factor models of King et al. (1998) and Simms et al. (2002), although some researchers argue that both models are in fact mis-specified (Shevlin, McBride, Armour, & Adamson, 2009). A recent meta-analysis on aggregated data from 40 different studies demonstrated that both the correlated four-factor numbing and dysphoria model provided superior model fit compared to alternative models across studies (Yufik & Simms, 2010). This meta-analysis also demonstrated that the dysphoria model outperformed the King et al. model in almost all subsamples.

The King et al. “emotional numbing” model contains four correlated factors: re-experiencing, (effortful) avoidance, emotional numbing, and arousal. In this model symptoms from the avoidance cluster (C) in *DSM-IV* are split into two different factors: active avoidance and emotional numbing. The avoidance factor consists of item C1 and C2. The emotional numbing factor includes items C3–C7 (see Table 2 for a legend of the symptom codes and for item allocation across models). The Simms et al. correlated four-factor “dysphoria” model contains the factors: re-experiencing, avoidance, dysphoria, and arousal. The re-experiencing factor is identical to the re-experiencing cluster in *DSM-IV* and contains all B cluster items. The avoidance factor is identical to the active avoidance factor in King et al. emotional numbing model, containing items C1 and C2. The dysphoria factor comprises items C3 through C7 as well as D1 through D3. The arousal factor comprises items D4 and D5. Recently, Elhai and colleagues (2011) proposed a model that places items D1 through D3 in a separate factor, and there is some evidence for enhanced model fit of this this five-factor hybrid of the numbing and dysphoria model for PTSD (Pietrzak, Tsai, Harpaz-Rotem, Whealin, & Southwick, 2012; Wang et al., 2011; Wang, Elhai, Dai, & Yao, 2012), as well as for acute stress disorder (Hansen, Armour, & Elklit, 2012).

As noted before, both the emotional numbing and dysphoria models differentiate between active or effortful avoidance and emotional numbing (Asmundson, Stapleton, & Taylor, 2004; Foa et al., 1995). King and colleagues constructed a separate factor for emotional numbing symptoms, whereas Simms and colleagues clustered the numbing symptoms under a broad dysphoria factor. The inclusion of this broad dysphoria factor in the Simms et al. model is theoretically appealing because it differentiates between specific and non-specific symptoms (King et al., 2006). Although Simms and colleagues did not test the fit of a hierarchical model that separates this non-specific factor from a higher-order PTSD factor that incorporates three sub-clusters (i.e., re-experiencing, effortful avoidance, arousal), the authors demonstrated that the dysphoria factor was highly correlated with indices of anxiety and depressive symptoms. This suggests that the dysphoria factor may reflect symptoms of general distress that PTSD has in common with other “distress” disorders (Clark, Watson, & Mineka, 1994). Indeed, using CFA Grant and colleagues demonstrated that PTSD could be distinguished from MDD and GAD and that intrusions, avoidance, and hypervigilance reflected lower-level PTSD symptom clusters,whereas dysphoria was best conceptualized as a higher-order factor common in PTSD, MDD, and GAD (Grant, Beck, Marques, Palyo, & Clapp, 2008). It should be noted, however, that a study by Marshall and colleagues demonstrated that this pattern of association is only present at the factor level. At the item level, correlations between the eight dysphoria items and an external measure of general distress are equally strong as the correlations of the PTSD items with measures of general distress (Marshall, Schell, & Miles, 2010).

So far, we have not come across studies that effectively separated PTSD-specific clusters from factors that may be associated with other psychiatric disorders in hierarchical models. Rather, studies that examined the fit of hierarchical models for PTSD either subsumed all factors under one higher-order PTSD factor or clustered avoidance and re-experiencing versus arousal and numbing symptoms in separate factors. On the whole, these studies did not provide much empirical support for the utility of hierarchical models to describe the symptom structure of PTSD (Asmundson et al., 2000; King et al., 1998; Taylor et al., 1998; Yufik & Simms, 2010). Therefore, the present study was aimed at examining the statistical validity of a hierarchical adaptation of the dysphoria model with clusters of re-experiencing, arousal, and effortful avoidance symptoms subsumed under a higher-order PTSD factor versus a dysphoria factor (see Fig. 1). To do so we tested the fit of this PTSD model on Clinician-Administered PTSD Scale (CAPS; Blake et al., 1995) data that were obtained from a heterogeneous trauma sample and compared it to the fit of alternative factor solutions.

**Fig. 1:**
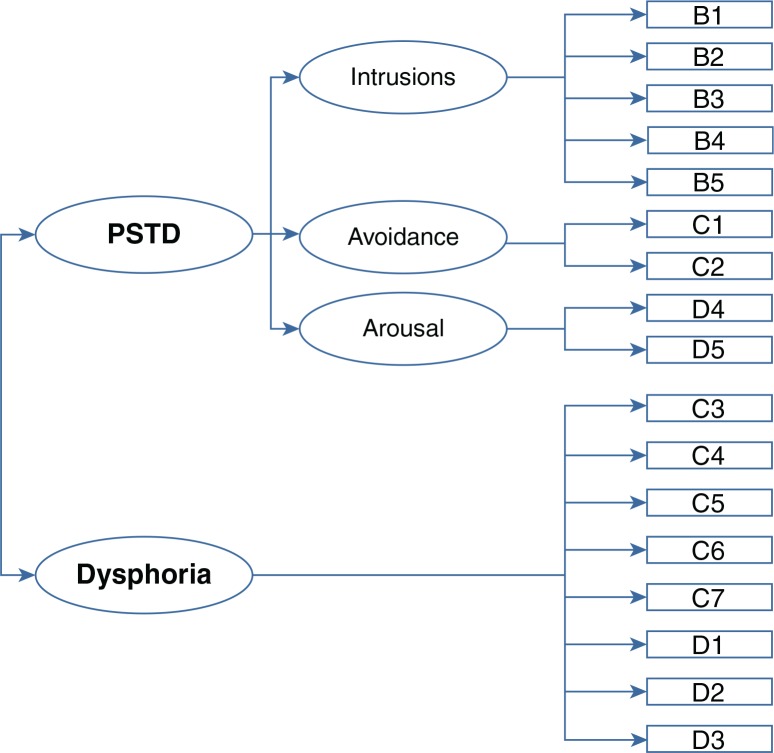
Hierarchical dysphoria model.

## Methods

### Participants & procedure

Data were acquired from treatment-seeking PTSD patients from military (*n*=115) and civilian (*n*=161) treatment facilities. Sites included the department of Military Mental Healthcare in Utrecht, the Netherlands, the Overwaal Centre for Anxiety Disorders in Lent, the Netherlands, and the Hendriks & Rooseboom Psychiatric and Psychotherapeutic Centre in Arnhem, the Netherlands. Data were collected from 2002 till 2011. Data were included from all patients who consented to have their data used for research. The CAPS was administered during the initial intake prior to the start of treatment. Participants were included in the study if they met *DSM-IV* criteria for PTSD, with a CAPS total score of ≥45 on the CAPS (Blake et al., 1995), which is indicative of (at least) moderate PTSD symptom severity according to the Dutch manual (Hovens, Luinge, & Van Minnen, 2005).

A total of 276 participants (48% female) were included in the analysis. Trauma exposure was assessed with the Life Event Checklist (LEC; Gray, Litz, Hsu, & Lombardo, 2004), which is routinely administered as part of the CAPS interview. Participants identified their index traumatic event as their “worst or most upsetting” traumatic event. Index traumatic events are reported in Table 1. The mean age of the sample was 36.3 years (SD=9.96, range 16–63). A total of 27% of the participants completed university or college or had at least some years of college/university education, 44.2% completed high school or had at least some year high school education, and 7.2% only completed elementary school. Mean elapsed time (years) since the traumatic experience (index event) was 11.9 (SD=11.04, range 0–54). The mean CAPS score was 73.3 (SD=16.78, range 45–125).

**Table 1:** Index traumatic events (*N*=276)

Trauma type	*n* (%)	Gender M/F	CAPS, *M* (SD)	Age, *M* (SD)
Combat-related	119 (43)	117/2	79.4 (17.35)	36 (7.8)
Sexual trauma	39 (14)	4/35	71.5 (19.77)	34 (12.2)
Interpersonal violence	47 (17)	11/36	68.3 (13.65)	35 (10.6)
Sexual trauma+violence	29 (11)	1/28	65.6 (13.03)	37 (10.3)
Accident	12 (4)	4/8	70.4 (16.65)	45 (11.3)
Other	27 (10)	7/20	66.7 (13.46)	39 (11.1)

### Measures

PTSD symptoms were assessed with the Dutch translation of the CAPS (Blake et al., 1995; Hovens et al., 2005). The CAPS is a 30-item structured interview that corresponds to *DSM-IV* criteria for PTSD. It contains 17 items that correspond to B, C, and D criteria for which frequency and intensity are rated on a five-point scale (0–4). Total PTSD severity is calculated as the sum of 17 symptom frequency and intensity scores. The CAPS has been shown to have excellent psychometric properties in a variety of populations (Weathers, Keane, & Davidson, 2001). The psychometric properties of the Dutch translation have been reported to be satisfactory (Hovens et al., 1994; Hovens et al., 2005).

### Statistical analyses

Data were multivariate non-normal distributed (Mardia's kurtosis coefficient=19.17; c.r.=6.173); therefore, CFA was conducted using maximum likelihood estimation with robust standard errors (MLR) in M-Plus v. 6.11 (Muthén & Muthén, 2011). MLR is robust to non-normality. It yields factor loadings identical to maximum likelihood estimation but adjusts the Chi-square and associated fit-indices and standard errors (Yuan & Bentler, 2000). Missing values were present for 3.8% of CAPS items. The maximum number of missing values for any individual was 2 out of 17 items, and the amount of missing values constituted just 0.002% of the total values. MLR estimation assumes missingness at random (MAR). Hereby, individuals with missing data are not excluded list-wise from the analyses, but all available data in the observed information matrix is used for parameter estimation, leading to unbiased parameter estimates.

Overall model fit was measured using the MLR χ^2^ statistic, which is equivalent to the Yuan-Bentler T*2 statistic, Root-Mean-Square-Error of Approximation (RMSEA), Standardized Root-Mean-Square Residual (SRMR), Comparative Fit Index (CFI), Tucker-Lewis Index (TLI), Akaike's Information Criterion (AIC), and the Bayesian Information Criterion (BIC). Cut-off scores of >0.95 for CFI and TLI, <0.07 for RMSEA, and < 0.08 for SRMR suggest good model fit (Byrne, 1989, 1991; Hu & Bentler, 1999; Steiger, 2007). The AIC and BIC are used to compare model fit of models with different amounts of parameters. Both indices represent a trade-off between model accuracy and model complexity (the BIC also corrects for the number of parameters). Lower values represent better fit. Additionally, when a model has a BIC value of 10 points less than another model, the odds that the model with the smaller BIC value is a better-fitting model would be 150:1, very strong evidence according to Raftery (1995).

Models that were investigated included: the current *DSM-IV* three-factor model (Model 1); the King et al. correlated four-factor emotional numbing model (King et al., 1998) (Model 2); the Simms et al. correlated four-factor dysphoria model (Model 3a) (Simms et al., 2002); a hierarchical four-factor dysphoria model with re-experiencing, avoidance and arousal clusters subsumed under a higher-order PTSD factor (Model 3b, see also Fig. 1); and the Elhai et al. correlated five-factor dysphoric arousal model (Model 4) (Elhai et al. 2011). Table 2 displays the item allocation across models.

**Table 2:** Item allocation in investigated models

	Model			
		
*DSM-IV*-TR PTSD symptoms	1	2	3a/b	4
B1. Intrusive thoughts	I	I	I	I
B2. Unpleasant dreams of the event	I	I	I	I
B3. Flashbacks	I	I	I	I
B4. Emotional reactivity	I	I	I	I
B5. Physiological reactivity	I	I	I	I
C1. Avoiding thoughts of trauma	Av	Av	Av	Av
C2. Avoiding reminders of trauma	Av	Av	Av	Av
C3. Inability to recall aspect of trauma	Av	N	D	N
C4. Loss of interest	Av	N	D	N
C5. Detachment	Av	N	D	N
C6. Restricted affect	Av	N	D	N
C7. Sense of foreshortened future	Av	N	D	N
D1. Sleep disturbance	Ar	Ar	D	DA
D2. Irritability	Ar	Ar	D	DA
D3. Difficulty concentrating	Ar	Ar	D	DA
D4. Hyper vigilance	Ar	Ar	Ar	AA
D5. Exaggerated startle response	Ar	Ar	Ar	AA

Note: AA=Anxious arousal; Ar=Arousal; Av=Avoidance; D=Dysphoria; DA=Dysphoric arousal; *I*=Intrusions; *N*=Numbing; Model 1=*DSM-IV*; Model 2=King et al. (1998) four-factor numbing; Model 3=Simms et al. (2002) four-factor dysphoria; Model 4=Elhai et al. (2011) five-factor dysphoric arousal.

## Results

Table 3 shows fit-indices of the models that were evaluated. CFI and TLI values were below cut-off in all models. All models provided better fit than the *DSM-IV* reference model (1). Variations of the dysphoria model slightly outperformed the numbing model. Additionally, although differences in fit-indices across models were small, the 10 point difference in BIC value between Simms et al. correlated (3a) and hierarchical dysphoria model (3b) should be considered as positive evidence that the latter is a better-fitting model (Raftery, 1995). Moreover, a corrected Chi-square difference test for nested models was non-significant [S-B scaled *χ* ^2^-diff (2, *N*=276)=0.64, *p*=0.73], suggesting that the more parsimonious nested hierarchical model (3b) should be retained over the inter-correlated four-factor dysphoria model (3a). As can be seen in Table 4, standardized factor loadings of CAPS items vary modestly across models. Items with poor factor loadings across models included C3, inability to recall aspects of trauma, and D1, sleep disturbances. However, removing these items from the analyses did not substantially improve model fit.

**Table 3:** Confirmatory factor analysis results: fit-indices of investigated models

Model	*χ* ^2^*	*df*	CFI	TLI	RMSEA	SRMR	AIC	BIC
1	291.632	116	0.688	0.634	0.074	0.078	20861.330	21056.832
2	251.926	113	0.753	0.703	0.067	0.070	20822.517	21028.879
3a	216.842	113	0.815	0.778	0.058	0.064	20789.496	20995.858
**3b**	**215.963**	**115**	**0.821**	**0.788**	**0.056**	**0.064**	**20786.377**	**20985.499**
4	218.912	109	0.805	0.756	0.060	0.064	20796.190	21017.034

Note: Model 1=*DSM-IV*; Model 2=King et al. (1998) correlated four-factor numbing; Model 3a=Simms et al. (2002) correlated four-factor dysphoria; Model 3b= hierarchical four-factor dysphoria; Model 4=Elhai et al. (2011) five-factor dysphoric arousal; *=MLR χ^2^ statistic, *p* < 0.001; AIC=Akaike's Information Criterion; BIC=Bayesian Information Criterion; CFI=Comparative Fit Index; RMSEA=Root-Mean-Square-Error of Approximation; SRMR=Standardized Root-Mean-Square Residual; TLI=Tucker-Lewis Index; Best fit indicated in bold-print.

* =MLR χ^2^ statistic, *p* < 0.001;

AIC=Akaike's Information Criterion; BIC=Bayesian Information Criterion; CFI=Comparative Fit Index; RMSEA=Root-Mean-Square-Error of Approximation; SRMR=Standardized Root-Mean-Square Residual; TLI=Tucker-Lewis Index; Best fit indicated in bold-print.

**Table 4:** Standardized factor loadings in models under investigation

	Model				
		
*DSM-IV*-TR PTSD symptoms	1	2	3a	3b*	4
B1. Intrusive thoughts	0.505	0.469	0.474	0.472	0.472
B2. Unpleasant dreams of the event	0.307	0.293	0.300	0.300	0.296
B3. Flashbacks	0.421	0.384	0.376	0.373	0.375
B4. Emotional reactivity	0.714	0.740	0.735	0.735	0.738
B5. Physiological reactivity	0.554	0.583	0.587	0.591	0.588
C1. Avoiding thoughts of trauma	0.331	0.688	0.598	0.613	0.605
C2. Avoiding reminders of trauma	0.124	0.389	0.448	0.437	0.443
C3. Inability to recall aspect of trauma	0.095	0.104	0.109	0.109	0.108
C4. Loss of interest	0.527	0.532	0.561	0.557	0.563
C5. Detachment	0.646	0.668	0.662	0.662	0.661
C6. Restricted affect	0.670	0.706	0.684	0.685	0.687
C7. Sense of foreshortened future	0.454	0.431	0.434	0.436	0.429
D1. Sleep disturbance	0.296	0.278	0.259	0.257	0.249
D2. Irritability	0.441	0.467	0.389	0.394	0.388
D3. Difficulty concentrating	0.373	0.322	0.407	0.406	0.369
D4. Hypervigilance	0.384	0.431	0.458	0.437	0.481
D5. Exaggerated startle response	0.239	0.287	0.640	0.620	0.610

Note: *Factor correlation between PTSD and Dysphoria=0,399 Model 1=*DSM-IV*; Model 2=King et al. (1998) correlated four-factor numbing; Model 3a=Simms et al. (2002) correlated four-factor dysphoria; Model 3b= hierarchical four-factor dysphoria; Model 4=Elhai et al. (2011) five-factor dysphoric arousal.

## Discussion

The present study examined the latent structure of PTSD symptoms in a mixed-trauma sample. Although the differences between fit-indices were small, the hierarchical dysphoria model fitted the data best. The proposed model that included clusters of arousal, intrusion, and effortful avoidance symptoms subsumed under a higher-order PTSD factor, and a separate dysphoria factor fitted the data (marginally) better than the original dysphoria model. Superior fit was also confirmed by a Chi-square difference test. The focus on a mixed-trauma sample in the present study suggests that the reported factor structure is fairly robust to inter-subject variation in type of trauma. Additionally, the results converge with previous studies (e.g., Baschnagel et al., 2005; Carragher, Mills, Slade, Teesson, & Silove, 2010; Olff, Sijbrandij, Opmeer, Carlier, & Gersons, 2009; Pietrzak, Goldstein, Malley, Rivers, & Southwick, 2010) and a recent meta-analysis (Yufik & Simms, 2010), as it demonstrated that the Simms et al. dysphoria model (Simms et al., 2002) performed better-albeit modestly-than the King et al. numbing model (King et al., 1998).

Item C3 (trauma-related amnesia) performed poorly across models. Therefore, these findings lend empirical support to McNally's (2009) proposal to remove the item from the diagnostic criteria for PTSD. Although removal of item C3 did not substantially improve model fit in the present study, other studies provide additional support for the removal of item C3 (Grant et al., 2008; McWilliams, Cox, & Asmundson, 2005; Olff et al., 2009). Item D1 (sleep problems) also displayed poor factor loadings across models. Again, removing the item did not improve model fit in the present study. This finding may be taken to imply that this item taps on a separate latent variable representing sleeping problems (King et al., 2009; Morina, et al., 2010), although we did not test the fit of such a model in the present study.

The present study underscores the validity of a demarcation between specific and non-specific (i.e., dysphoria) PTSD symptoms. This view is further supported by the proposed reformulation of PTSD by Brewin and colleagues (Brewin, Lanius, Novac, Schnyder, & Galea, 2009). Separating PTSD from dysphoria symptoms provides the possibility to examine the etiology and pathogenesis of PTSD within an integrative hierarchical model for mood and anxiety disorders (Mineka, Watson, & Clark, 1998; Simms et al., 2002; see also, Watson, 2005), which postulates that all mood and anxiety disorders share a higher-order component of general distress, versus lower-level factors underlying specific (clusters of) mood and anxiety disorders. PTSD is proposed to share a broad, non-specific factor with other “distress” and emotional/internalizing disorders but is expected to be distinguishable by one or more specific lower-level components (Watson, 2005). Accordingly, a recent meta-analysis demonstrated that dysphoria is more strongly correlated to indices of depression, anxiety, panic, and substance abuse than the intrusions, avoidance, and hypervigilance factors (Gootzeit & Markon, 2011).

Grant and colleagues ( 2008) observed that PTSD, MDD, and GAD were highly correlated disorders in a sample of MVA victims and that a higher-order dysphoria factor accounted for these correlations. Also, Gros and colleagues (2010) demonstrated that emotional numbing and dysphoria symptoms increased the likelihood of meeting diagnostic criteria for (comorbid) MDD in PTSD patients. Moreover, a recent study by Wolf et al. (2010) confirmed that PTSD shares a common genetic risk factor with MDD, GAD, panic disorder, and DD. It should be noted, however, that the dysphoria factor is not uniquely associated with external measures of general distress. A recent study by Armour and colleagues demonstrated that a significant amount of variance in the dysphoria factor is accounted for by MDD and GAD, but that the variance in intrusive symptoms was more strongly affected by statistically controlling for the presence for GAD (after controlling for MDD) than the variance in dysphoria items (Armour, McBride, Shevlin, & Adamson, 2011).

Marshall and colleagues (2010) confirmed that the dysphoria factor is most strongly related to external measures of depressive symptoms and anxiety. More importantly, however, they observed that at the item level, correlations of general distress with external measures were not stronger for the dysphoria items than for the PTSD items in Simms et al. model. To account for their findings, the authors posited that the dysphoria factor might actually tap on impaired functioning (analogous to Criterion F in *DSM-IV*) as caused by the remaining PTSD items. An alternative explanation is that the items in the dysphoria cluster actually tap on aspects of (trait) neuroticism/negative affectivity (N/NA). N/NA incorporates negative emotional states such as fear, anger, sadness, guilt, and disgust (Clark et al., 1994; Watson, 2005, p. 525). Although N/NA is not specific to mood and anxiety disorders (Mineka et al., 1998), prospective research has demonstrated that N/NA composes a particularly salient feature in the etiology of distress disorders like PTSD (e.g., Parslow, Jorm, & Christensen, 2006; Rademaker, van Zuiden, Vermetten, & Geuze, 2011) and MDD (Christensen & Kessing, 2006).

The relevance of effectively differentiating between PTSD symptoms and general distress becomes all the more apparent when we examine the proposed revisions for PTSD in *DSM-V*. The proposed revision indicates a four-factor model that includes re-experiencing, active avoidance, negative mood/cognition, and hyperarousal factors. Although the specification of a separate active avoidance cluster corresponds to the extant body of CFA results, the negative emotionality and hyperarousal cluster closely resemble the C and D criteria in *DSM-IV*. Therefore, it seems likely that the proposed diagnostic criteria for PTSD in DSM-V will continue to be a source for debate. More importantly, however, the inclusion of items in DSM-V to assess “persistent blame of self and others” and a “pervasive negative emotional state”, which appear to tap on (trait) N/NA, can be expected to further increase the overlap of PTSD with other distress disorders. If so, this would inadvertently and somewhat paradoxically strengthen claims for a need to change the nosologic system of DSM (e.g., Maser et al., 2009; Watson, 2005).

Marshall et al. (2010) proposed that the dysphoria factor might best be relabeled to reflect that it represents a multifacetted cluster of items associated with impaired functioning (e.g., sleeping problems, difficulty concentrating, reduced affect, loss of interest). Indeed, recent data suggest that the dysphoria factor may comprise multiple aspects of impaired functioning. Studies have demonstrated that the dysphoria factor includes items that can be subsumed under (sub) factors for emotional numbing and sleep disturbances (King et al., 2009; Morina et al., 2010). Additionally, the dysphoria factor contains items pertaining to anhedonia and irritability, and there is evidence that both anhedonia and emotional numbing represent distinct pathogenic dimensions (Kashdan, Elhai, & Frueh, 2006). Moreover, since hostility has been reported to be a key feature in PTSD patients (Orth & Wieland, 2006), which is not specific to PTSD but occurs in various distress disorders (Moscovitch, McCabe, Antony, Rocca, & Swinson, 2008; Perlis et al., 2009), hostility could be a distinct sub-factor underlying the general distress/dysphoria cluster. Therefore, “lumping together” different symptom clusters into one dysphoria/general distress factor is inconsistent with evidence supporting the distinctness of these clusters (Palmieri, Weathers, Difede, & King, 2007, p. 339), and additional research into the dimensions underlying the dysphoria construct is recommended.

The results of the present study have to be viewed in light of a number of limitations. First, the observed fit-indices were inconsistent. Whereas the RMSEA suggested good fit (< 0.07) in all models (except the *DSM-IV* model), the TLI and CFI values indicated poor model fit. The CFI values were lower in the present study than in other studies that used the CAPS (Blake et al., 1995) to assess PTSD symptoms (Buckley et al., 1998; Palmieri et al., 2007). The CFI is affected by the average size of correlations in the data (Kenny, 2012), which may have been lower in the present sample as compared to results from previous studies in more homogenous samples.^1^ For instance, whereas previous studies examined fairly homogenous trauma samples like female victims of domestic violence (Elhai et al., 2011), earthquake survivors (Wang et al., 2011; Wang et al., 2012), or military veterans (King et al., 1998), the present study included a range of trauma types including sexual trauma, combat, and interpersonal violence. Nevertheless, the CFI, TLI, and BIC values of the hierarchical adaptation of the Simms et al. dysphoria model were superior to those of competing models.

A second limitation pertains to the fact that the present sample consisted of treatment-seeking individuals diagnosed with PTSD only. Therefore, additional research is needed to determine whether results can be generalized to community-dwelling trauma-exposed individuals. Relatedly, it is possible that selecting a sample of individuals diagnosed with PTSD exclusively, rather than a sample of individuals meeting *DSM-IV* criterion A (and not necessarily meeting all diagnostic criteria for PTSD), as was the case in most previous studies, may have biased the results. Indeed, a factorial invariance study in US veterans suggests that although model fit may be equal across groups (PTSD vs. criterion A), there may exists (subtle) differences between (treatment-seeking) PTSD patients and trauma-controls in the way they perceive and respond to PTSD questionnaires (McDonald, Beckham, Morey, & Calhoun, 2009).

## Conclusion

Uncovering the latent structure of PTSD is important in identifying distinct aspects of PTSD symptomatology and sources of comorbidity with order disorders. The present study demonstrates once more that the three-factor solution in *DSM-IV*-TR is untenable and that a model that separates core PTSD symptoms from items associated with general distress or impaired functioning, which are shared with other psychiatric disorders, provides better fit to the data. Future studies examining the (neurobiological) correlates of the factors described in this and previous studies, as well as studies into dimensions that may underlie the general distress/dysphoria factor, would help to further our understanding of the pathogenesis of PTSD and other distress disorders.

## Footnotes

1 Full results for the models in this study, including details on correlations, may be obtained from the first author.

## References

[CIT0001] American Psychiatric Association (2000). Diagnostic and statistical manual of mental disorders.

[CIT0002] Anthony J. L, Lonigan C. J, Hecht S. A (1999). Dimensionality of posttraumatic stress disorder symptoms in children exposed to disaster: Results from confirmatory factor analyses. Journal of Abnormal Psychology.

[CIT0003] Armour C, McBride O, Shevlin M, Adamson G (2011). Testing the robustness of the dysphoria factor of the Simms et al. (2002) model of posttraumatic stress disorder. Psychological Trauma: Theory, Research, Practice, and Policy.

[CIT0004] Asmundson G. J, Frombach I, McQuaid J, Pedrelli P, Lenox R, Stein M. B (2000). Dimensionality of posttraumatic stress symptoms: A confirmatory factor analysis of DSM-IV symptom clusters and other symptom models. Behaviour Research and Therapy.

[CIT0005] Asmundson G. J, Stapleton J. A, Taylor S (2004). Are avoidance and numbing distinct PTSD symptom clusters?. Journal of Traumatic Stress.

[CIT0006] Asmundson G. J, Wright K. D, McCreary D. R, Pedlar D (2003). Post-traumatic stress disorder symptoms in United Nations peacekeepers: An examination of factor structure in peacekeepers with and without chronic pain. Cognitive Behaviour Therapy.

[CIT0007] Baschnagel J. S, O'Connor R. M, Colder C. R, Hawk L. W (2005). Factor structure of posttraumatic stress among Western New York undergraduates following the September 11th terrorist attack on the World Trade Center. Journal of Traumatic Stress.

[CIT0008] Blake D. D, Weathers F. W, Nagy L. M, Kaloupek D. G, Gusman F. D, Charney D. S, Keane T. M (1995). The development of a Clinician-Administered PTSD Scale. Journal of Traumatic Stress.

[CIT0009] Brewin C. R, Lanius R. A, Novac A, Schnyder U, Galea S (2009). Reformulating PTSD for DSM-V: Life after Criterion A. Journal of Traumatic Stress.

[CIT0010] Buckley T. C, Blanchard E. B, Hickling E. J (1998). A confirmatory factor analysis of posttraumatic stress symptoms. Behaviour Research and Therapy.

[CIT0011] Byrne B. M (1989). A primer of LISREL: Basic applications and programming for confirmatory factor analytic models.

[CIT0012] Byrne B. M (1991). The Maslach Burnout Inventory: Validating factorial structure and invariance across intermediates, secondary, and university educators. Multivariate Behavioral Research.

[CIT0013] Carragher N, Mills K, Slade T, Teesson M, Silove D (2010). Factor structure of posttraumatic stress disorder symptoms in the Australian general population. Journal of Anxiety Disorders.

[CIT0014] Christensen M. V, Kessing L. V (2006). Do personality traits predict first onset in depressive and bipolar disorder?. Nordic Journal of Psychiatry.

[CIT0015] Clark L. A, Watson D, Mineka S (1994). Temperament, personality, and the mood and anxiety disorders. Journal of Abormal Pychology.

[CIT0016] Deering C. G, Glover S. G, Ready D, Eddleman H. C, Alarcon R. D (1996). Unique patterns of comorbidity in posttraumatic stress disorder from different sources of trauma. Comprehensive Psychiatry.

[CIT0017] Dragan M, Lis-Turlejska M, Popiel A, Szumial S, Dragan W. L (2012). The validation of the Polish version of the Posttraumatic Diagnostic Scale and its factor structure. European Journal of Psychotraumatology.

[CIT0018] Elhai J. D, Biehn T. L, Armour C, Klopper J. J, Frueh B. C, Palmieri P. A (2011). Evidence for a unique PTSD construct represented by PTSD's D1–D3 symptoms. Journal of Anxiety Disorders.

[CIT0019] Foa E. B, Riggs D. S, Gershuny B. S (1995). Arousal, numbing, and intrusion: symptom structure of PTSD following assault. American Journal of Psychiatry.

[CIT0020] Gootzeit J, Markon K (2011). Factors of PTSD: Differential specificity and external correlates. Clinical Psychology Review.

[CIT0021] Grant D. M, Beck J. G, Marques L, Palyo S. A, Clapp J. D (2008). The structure of distress following trauma: Posttraumatic stress disorder, major depressive disorder, and generalized anxiety disorder. Journal of Abnormal Psychology.

[CIT0022] Gray M. J, Litz B. T, Hsu J. L, Lombardo T. W (2004). Psychometric properties of the life events checklist. Assessment.

[CIT0023] Gros D. F, Simms L. J, Acierno R (2010). Specificity of posttraumatic stress disorder symptoms: an investigation of comorbidity between posttraumatic stress disorder symptoms and depression in treatment-seeking veterans. Journal of Nervous and Mental Disease.

[CIT0024] Hansen M, Armour C, Elklit A (2012). Assessing a dysphoric arousal model of acute stress disorder symptoms in a clinical sample of rape and bank robbery victims. European Journal of Psychotraumatology.

[CIT0025] Hovens J. E, Luinge B. A, Van Minnen A (2005). Het klinisch interview voor PTSS: Handleiding *[Dutch manual for the clinician adminstered PTSD scale]*.

[CIT0026] Hovens J. E, van der Ploeg H. M, Klaarenbeek M. T, Bramsen I, Schreuder J. N, Rivero V. V (1994). The assessment of posttraumatic stress disorder with the clinician administered PTSD scale: Dutch results. Journal of Clinical Psychology.

[CIT0027] Hu L.-T, Bentler P. M (1999). Cutoff criteria for fit indexes in covariance structure analysis: Conventional criteria versus new alternatives. Structural Equation Modeling.

[CIT0028] Kashdan T. B, Elhai J. D, Frueh B. C (2006). Anhedonia and emotional numbing in combat veterans with PTSD. Behaviour Research and Therapy.

[CIT0029] Kenny D. A (2012). Measuring model fit.

[CIT0030] King D. W, Leskin G. A, King L. A, Weathers F. W (1998). Confirmatory factor analysis of the clinician-administered PTSD scale: Evidence for the dimensionality of posttraumatic stress disorder. Psychological Assessment.

[CIT0031] King D. W, Orazem R. J, Lauterbach D, King L. A, Hebenstreit C. L, Shalev A. Y (2009). Factor structure of posttraumatic stress disorder as measured by the impact of event scale-revised: Stability across cultures and time. Psychological Trauma: Theory, Research, Practice, and Policy.

[CIT0032] King L. A, King D. W, Orazem R. J (2006). Research on the latent structure of PTSD. PTSD Research Quarterly.

[CIT0033] Marshall G. N, Schell T. L, Miles J. N (2010). All PTSD symptoms are highly associated with general distress: ramifications for the dysphoria symptom cluster. Journal of Abnormal Psychology.

[CIT0034] Maser J. D, Norman S. B, Zisook S, Everall I. P, Stein M. B, Schettler P. J, Judd L. L (2009). Psychiatric nosology is ready for a paradigm shift in DSM-V. Clinical Psychology: Science and Practice.

[CIT0035] McDonald S. D, Beckham J. C, Morey R. A, Calhoun P. S (2009). The validity and diagnostic efficiency of the Davidson Trauma Scale in military veterans who have served since September 11th, 2001. Journal of Anxiety Disorders.

[CIT0036] McNally R. J (2009). Can we fix PTSD in DSM-V?. Depression and Anxiety.

[CIT0037] McWilliams L. A, Cox B. J, Asmundson G. J (2005). Symptom structure of posttraumatic stress disorder in a nationally representative sample. Journal of Anxiety Disorders.

[CIT0038] Mineka S, Watson D, Clark L. A (1998). Comorbidity of anxiety and unipolar mood disorders. Annual Review of Psychology.

[CIT0039] Morina N, Böhme H. F, Ajdukovic D, Bogic M, Franciskovic T, Galeazzi G. M, Priebe S, … (2010). The structure of post-traumatic stress symptoms in survivors of war: Confirmatory factor analyses of the impact of event scale-revised. Journal of Anxiety Disorders.

[CIT0040] Moscovitch D. A, McCabe R. E, Antony M. M, Rocca L, Swinson R. P (2008). Anger experience and expression across the anxiety disorders. Depression and anxiety.

[CIT0041] Muthén, Muthén (2011). Mplus (Version 6.11).

[CIT0042] Olff M, Sijbrandij M, Opmeer B. C, Carlier I. V, Gersons B. P (2009). The structure of acute posttraumatic stress symptoms: ‘Reexperiencing’, ‘active avoidance’, ‘dysphoria’, and ‘hyperarousal’. Journal of Anxiety Disorders.

[CIT0043] Orth U, Wieland E (2006). Anger, hostility, and posttraumatic stress disorder in trauma-exposed adults: A meta-analysis. Journal of Consulting and Clinical Psychology.

[CIT0044] Palmieri P. A, Weathers F. W, Difede J, King D. W (2007). Confirmatory factor analysis of the PTSD checklist and the clinician-administered PTSD scale in disaster workers exposed to the World Trade Center Ground Zero. Journal of Abnormal Psychology.

[CIT0045] Parslow R. A, Jorm A. F, Christensen H (2006). Associations of pre-trauma attributes and trauma exposure with screening positive for PTSD: Analysis of a community-based study of 2085 young adults. Psychological Medicine.

[CIT0046] Perlis R. H, Fava M, Trivedi M. H, Alpert J, Luther J. F, Wisniewski S. R, John Rush A (2009). Irritability is associated with anxiety and greater severity, but not bipolar spectrum features, in major depressive disorder. Acta Psychiatrica Scandinavica.

[CIT0047] Pietrzak R. H, Goldstein M. B, Malley J. C, Rivers A. J, Southwick S. M (2010). Structure of posttraumatic stress disorder symptoms and psychosocial functioning in Veterans of Operations Enduring Freedom and Iraqi Freedom. Psychiatry Research.

[CIT0048] Pietrzak R. H, Tsai J, Harpaz-Rotem I, Whealin J. M, Southwick S. M (2012). Support for a novel five-factor model of posttraumatic stress symptoms in three independent samples of Iraq/Afghanistan veterans: A confirmatory factor analytic study. Journal of Psychiatric Research.

[CIT0049] Rademaker A. R, van Zuiden M, Vermetten E, Geuze E (2011). Type D personality and the development of PTSD symptoms: A prospective study. Journal of Abnormal Psychology.

[CIT0050] Raftery A. E (1995). Bayesian model selection in social research. Sociological Methodology.

[CIT0051] Rosen G. M, Lilienfeld S. O (2008). Posttraumatic stress disorder: An empirical evaluation of core assumptions. Clinical Psychology Review.

[CIT0052] Shevlin M, McBride O, Armour C, Adamson G (2009). Reconciling the differences between the King et al. (1998) and Simms et al. (2002) factor models of PTSD. Journal of Anxiety Disorders.

[CIT0053] Simms L. J, Watson D, Doebbeling B. N (2002). Confirmatory factor analyses of posttraumatic stress symptoms in deployed and nondeployed veterans of the Gulf War. Journal of Abnormal Psychology.

[CIT0054] Spitzer R. L, First M. B, Wakefield J. C (2007). Saving PTSD from itself in DSM-V. Journal of Anxiety Disorders.

[CIT0055] Steiger J. H (2007). Understanding the limitations of global fit assessment in structural equation modeling. Personality and Individual Differences.

[CIT0056] Taylor S, Koch W. J, Kuch K, Crockett D. J, Passey G (1998). The structure of posttraumatic stress symptoms. Journal of Abnormal Psychology.

[CIT0057] Wang L, Zhang J, Shi Z, Zhou M, Li Z, Zhang K, Elhai J. D, … (2011). Comparing alternative factor models of PTSD symptoms across earthquake victims and violent riot witnesses in China: Evidence for a five-factor model proposed by Elhai et al. (2011). Journal of Anxiety Disorders.

[CIT0058] Wang M, Elhai J. D, Dai X, Yao S (2012). Longitudinal invariance of posttraumatic stress disorder symptoms in adolescent earthquake survivors. Journal of Anxiety Disorders.

[CIT0059] Watson C. G, Kucala T, Juba M, Manifold V, Anderson P. E, Anderson D (1991). A factor analysis of the DSM-III post-traumatic stress disorder criteria. Journal of Clinical Psychology.

[CIT0060] Watson D (2005). Rethinking the mood and anxiety disorders: A quantitative hierarchical model for DSM-V. Journal of Abnormal Psychology.

[CIT0061] Weathers F. W, Keane T. M, Davidson J. R (2001). Clinician-administered PTSD scale: A review of the first ten years of research. Depression and Anxiety.

[CIT0062] Wolf E. J, Miller M. W, Krueger R. F, Lyons M. J, Tsuang M. T, Koenen K. C (2010). Posttraumatic stress disorder and the genetic structure of comorbidity. Journal of Abnormal Psychology.

[CIT0063] Yuan K. H, Bentler P. M, Sobel M. E, Becker M. P (2000). Three likelihood-based methods for mean and covariance structure analysis with nonnormal missing data. Sociological methodology.

[CIT0064] Yufik T, Simms L. J (2010). A meta-analytic investigation of the structure of posttraumatic stress disorder symptoms. Journal of Abnormal Psychology.

